# Competency‐based assessment in nutrition education: A systematic literature review

**DOI:** 10.1111/jhn.12946

**Published:** 2021-09-19

**Authors:** Sarah O'Donovan, Claire Palermo, Lisa Ryan

**Affiliations:** ^1^ Department of Sport, Exercise and Nutrition Galway‐Mayo Institute of Technology Galway Ireland; ^2^ Department of Nutrition Dietetics and Food, Monash University Melbourne VIC Australia

**Keywords:** assessment, competency, nutrition education

## Abstract

**Background:**

A suitably prepared and qualified nutrition and dietetics workforce is part of the solution to combating the burden of disease. Competency‐based assessment is a key part of the education of future workforces. Although there has been recent attention on competency‐based assessment in dietetics, there is little exploration of competency‐based education for the preparation of nutritionists. The present study aimed to understand how competency‐based assessment is implemented and evaluated in nutrition education.

**Methods:**

A systematic literature review was carried out according to PRISMA guidelines. Four databases were initially searched in February 2020 using key words related to competenc* in combination with *nutrition* or *dietetic* and their synonyms. An updated search was completed again in March 2021. Studies that met eligibility criteria where the focus was on nutrition and involved a method of competency‐based assessment were synthesised narratively.

**Results:**

From a total of 6262 titles and abstracts, six studies on competency assessment in nutrition education were identified. The assessments focused on the development of key skills, including motivational interviewing and nutrition assessment, changes to knowledge and attitudes on food and culture, and self‐perceived development of communication, collaboration, management, advocacy, scholarship and professional capabilities. No studies were found that assessed promotion of health and wellbeing or the food chain competencies.

**Conclusions:**

The lack of research in competency‐based assessment must be addressed to ensure we are effectively preparing future nutritionists for work such that they can impact health outcomes.

## Graphical abstract

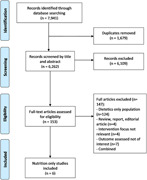

## INTRODUCTION

Dietary risk factors account for much of the burden of ill health and death across the world.^1^ A suitably prepared and qualified nutrition and dietetics workforce is part of the solution to combating the improvements needed to address this burden of disease. Competency‐based education frameworks ensure the workforce meets current and future health and, in this context, nutrition needs.^2^ Competency standards or frameworks are a key part of competency‐based education. Competency‐standards describe the skills and abilities required of the workforce which guide curricula supporting the preparation of graduates for the workforce. Competency‐based assessment (CBA) involves measurement of a student's competence by analysing their performance and achievements against competence standards^3^ or the processes used to measure a student's ability to apply theory into practice,^4^ and is a key part of educating future workforces. Although there has been recent attention on CBA in dietetics,^4^, ^5^, ^6^, ^7^, ^8^ there is little exploration of competency‐based education for the preparation of nutritionists.

Nutritionists are nutrition science experts. In Ireland and the UK, the role of a nutritionist is to provide scientific evidence‐based information on food and healthy eating to individuals or the larger public outside of hospital settings,^9^ mainly working in preventative roles, across multiple sectors, predominately with healthy people and populations.^10^ Although the definition and function vary across the world, primarily as a result of a lack of regulation of the title ‘Nutritionist’, in the UK, nutritionists can apply for registration. Implicit in this registration system the five core nutritionist competency areas are science, food/feed chain, professional conduct, social/behavioural, and health/wellbeing.^11^ There is an accreditation system based on these five core competencies and set accreditation standards to educate nutritionists. This is a voluntary accreditation system carried out by the Association for Nutrition (AfN), for both individuals and courses. The process for accreditation includes mapping a program's curriculum and modules to these core competencies and the standards for ethics and conduct. Evidence of assessment for each competence is required to show that the level of competencies acquired upon completion of the degree meet the high standards expected. Registering as a nutritionist with the AfN provides assurance to future employers and clients that high standards of competence and professionalism have been met,^9^ as well as offering a guarantee to the public about the quality of the nutritionist. Yet, there has been no examination of the CBA processes or methods used in the preparation of nutritionists for the workforce in the UK or beyond.

Ensuring future nutritionist graduates are adequately prepared for the workforce is essential to improve population health and nutrition. Understanding how CBA of nutritionists is carried out will provide insight into the adequacy of this preparation. Therefore, the primary aim of this systematic review is to understand how CBA is implemented and evaluated in the preparation of nutritionists internationally.

## METHODS

### Search strategy

A systematic review (PROSPERO registration: CRD42021237951) was carried out according to the Preferred Reporting Items for Systematic Reviews and Meta‐Analyses (PRISMA) guidelines.^12^ The PICO (Participant, Intervention, Comparator, Outcome) framework was used to define the research question: How is competence assessed in nutritionist training? A systematic literature search was carried out in February 2020 using the databases: PubMed, CINAHL Complete, Web of Knowledge and Science Direct. Following an initial search of the databases to identify key search terms, a comprehensive search was completed to gather all relevant studies. Key words used included competenc* in combination with *nutrition* or *dietetic* and their relevant synonyms. Searches were exported for screening (see Supporting information, Appendix S1). An updated search was carried out in March 2021 to capture any new research meeting the PICO criteria.

### Study selection

A three‐stage screening process was carried out. The first and second stages were completed by the first investigator (SOD) to remove all duplicates and review titles, and then abstracts. Over 10% of abstracts were screened by all investigators (SOD, CP, LR) and differences were discussed to reach agreement. The third stage involved all authors reviewing the studies in full against the criteria (Table 1) to identify those eligible for inclusion. Any disagreement about the eligibility of studies was resolved by consensus between three investigators. Only empirical research studies of any study design, focused on implementation and evaluation of competency assessment in nutrition education, and written in English, were included. Studies focussed on CBA in dietetics education, opinion pieces or editorials were excluded.

**Table 1:** Inclusion and exclusion criteria

Criteria	Inclusion	Exclusion
Population	Humans Adults ≥ 18 years Students/graduates of nutrition degree	Animals Children Courses on nutritional therapy Degree programs < third level Dietetics only
Intervention	Implementation of competency‐based assessment Evaluation of competency‐based assessment	No mention of competency‐based assessment or its evaluation
Comparator	NA	
Outcome	Opinion on competency‐based assessment Analysis of effectiveness/pre‐ and post‐analysis of competency	No measure of competency analysis
Study design	Primary empirical research (all study designs) English language only	All other study designs and non‐English publications

Abbreviation: NA, not applicable.

### Data synthesis

A data extraction template was developed and included authors, year of publication, location, sample population, competency assessment method, method of evaluating the competency assessment, results and competency assessed based on AfN Competency Standards (see Supporting information, Appendix S2). The Critical Appraisal Skills Program (CASP) Qualitative Research Checklist (10 questions),^13^ was used to assess the quality of the qualitative studies included. The CASP Cohort Study Checklist (12 questions)^13^ was used to assess the quality of the quantitative studies. A three‐point rating system of low, medium or high indicated the quality of each article. Studies were rated as low (≤40%) ≤ 4/10 or 5/12; low–moderate methodological issues), medium (between 40% and 70%; minor methodological issues) and high (≥70%) (≥7/10 or 9/12; high–robust). Extracted data were synthesised using a narrative approach where all elements of each of the included studies were summarised narratively.^14^ All studies, regardless of quality, were weighted equally in the synthesis.

## RESULTS

**Figure 1:**
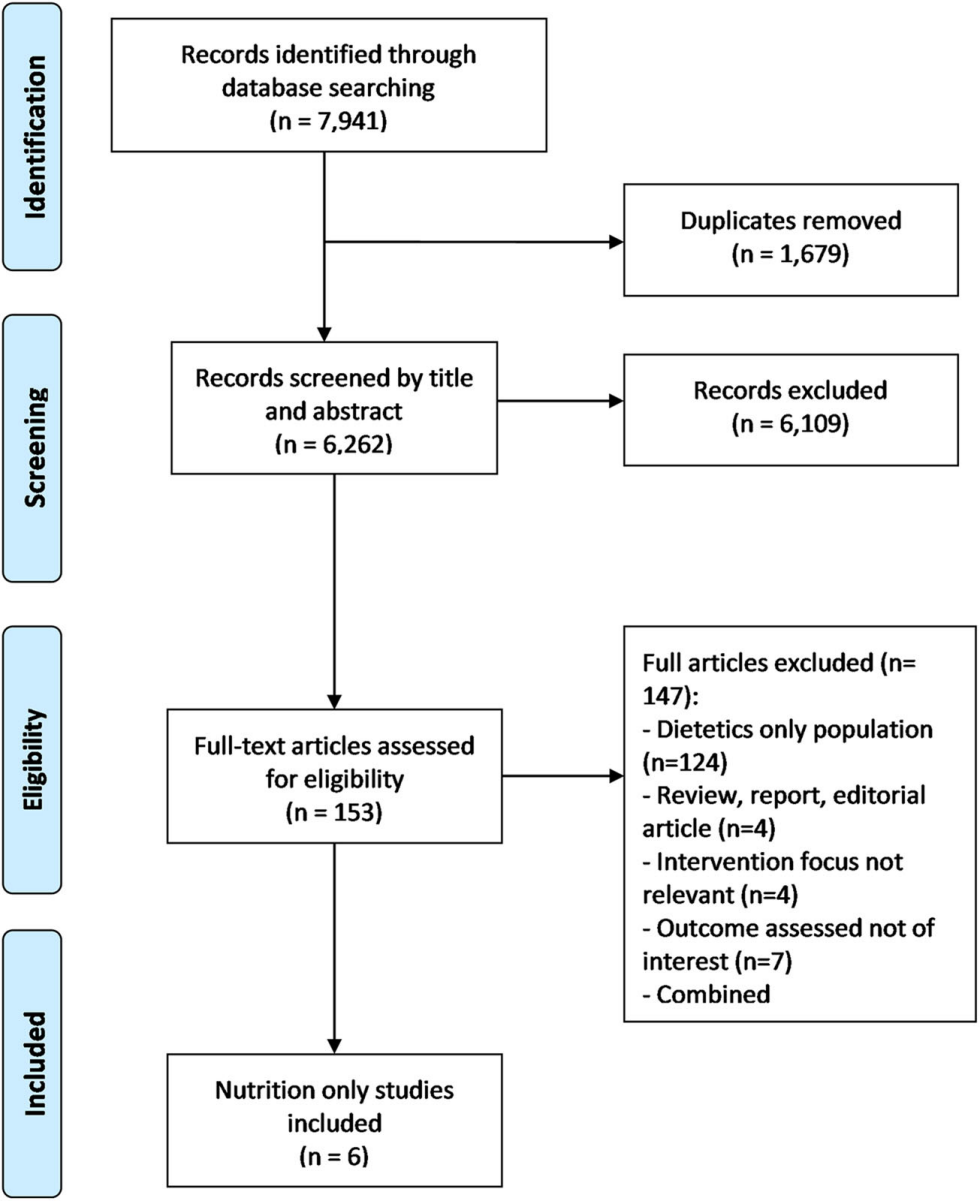
PRISMA flow diagram illustrating the process of reviewing and identifying eligible studies which meet the outlined PICO (Participant, Intervention, Comparator, Outcome) criteria

In total, 7941 records were identified through the database searches. After removal of 1679 duplicates, six nutrition only studies were eligible for inclusion (Figure 1). Of the six included nutrition studies, four were qualitative^15^, ^16^, ^17^, ^18^ and two were quantitative.^19^, ^20^ Mapping these to the AfN areas of core competency highlighted that a different competency area was assessed within each of the studies. These included, cultural competence,^17^, ^19^, ^20^ behaviour change counselling,^15^ self‐reflection^16^ and nutritional assessment skills.^18^ The Feed/Food Chain (C2) and the Health/Wellbeing (C4) competencies were not covered by any of the included studies (Table 2). The methods used to gain feedback on the assessment approaches included face‐to‐face interviews, portfolio analysis, pre‐ and post‐analysis questionnaires, and video assessments. Of the six studies analysed using the CASP checklists, four were of high quality and two were of medium quality as a result of a lack of details surrounding the research design and recruitment strategies

**Table 2:** Included studies mapped against the Association for Nutrition (AfN) core competencies

AfN core competency	Competency sub‐group covered
Science (C1)	Garcia de Diego et al.^18^: *CC1g – Nutrient analysis: calculating nutrient contents of foods, feeds and diets of an individual or group of individuals or animals, justifying choice of a method of dietary assessment for a specific stated purpose*
	Sabatini et al.^17^: *CC1o – Prepare, process, interpret and present data, using appropriate qualitative and quantitative techniques, statistical programmes, spreadsheets and programs for presenting data visually*
Food/feed chain (C2)	
Social/behavioural (C3)	Bauer and Bai^19^ and McCartan et al.^20^: *CC3d – Religious and cultural beliefs and practices that impact on food, nutrition and health*
	Simper et al.^15^: *CC3f – Theories and application of methods of improving health, behaviour and change for either human or animal systems*
Health/wellbeing (C4)	
Professional conduct (C5)	Marais et al.^16^: *CC5c – Legal context of nutrition practice; including current relevant legislation and guidelines to providing information to individuals*

### Synthesis of results

The sample populations of the studies ranged from 20 to 52 students of nutrition specific degrees (Table 3). The geographical locations of the studies included Africa,^16^ Europe,^15^, ^16^, ^18^ North America,^19^ Australia^20^ and South America.^17^ In five studies, performance was assessed using objective (*n* = 5) tests of knowledge.^15^, ^17^, ^18^, ^19^, ^20^ Marais et al.^16^ was the only study that implemented a subjective assessment using self‐reflection via an interview.

**Table 3:** Summary of the main information extracted from the studies included in the systematic literature review

Author, date, setting	Sample population	CBA method	Method of evaluating CBA	Main results	Quality analysis (CASP) rating^13^
Simper et al.,^15^ Sheffield Hallam University, UK	52 students from final year undergraduate nutrition cohort	Behaviour change counselling competence following 5 × 3 h motivational interviewing training sessions, two recorded practice sessions (at beginning and end) with an actor portraying a client and one feedback session	Video assessment at baseline and follow‐up after training sessions	Significant improvement seen for all six of the behaviours coded. A decrease in closed questions and non‐adherent behaviour and an increase in open questions, complex and simple reflections, etc. Talk time ratio also improved with nutritionists allowing more time for the client to talk and elaborate Baseline proficiency was seen to be below where it should be for all clinician‐behaviour counts. At follow‐up, these were above beginning proficiency showing an improvement for all students to being on the way to becoming competent. Improvement was significant for all counts with *p* < 0.001	8/10
Marais et al.,^16^ Universities in Norway, Uganda and South Africa	20 students completing master's degree in Nutrition in universities in Norway, South Africa and Uganda, 16 were female, Mean age (SD) was 30.2 (6.0) years	Self‐reflection interviews following 18‐week NOMA track module	Interviews themed by CanMEDS competency framework	Themes suggest students developed competency:	5/10
				As communicators As collaborators, health and nutrition professional effectively work within a team to achieve optimal service‐user care As managers As health and nutrition advocates As scholars As professionals	
Sabatini et al.,^17^ UNIFESP, Sao Paulo, Brazil	29 (of 49) students majoring in nutrition submitted portfolios for analysis 11/29 attended focus groups evaluating the portfolio	Construction of portfolio about food and culture	Focus group and analysis of portfolios one year after development	Significant manifestation of how the portfolio stimulated curiosity and autonomy for learning, and fostered greater interest in food and culture Greater sociocultural appreciation of eating observed Students perceived the creation of the portfolio as an exciting and engaging process, although demanding Similarity between messages recorded in portfolios and opinions expressed in focus groups conducted after 1 year, suggesting portfolio had a long‐term reach, promoting reflection and autonomy	7/10
Garcia de Diego et al.,^18^ University of Navarra, Spain	30 volunteers: 14 students of master's degree in Food Science, Nutrition and Metabolism; 12 health sciences graduates; and 4 PhDs in nutritional sciences	Ability to use new computer assisted instruction (CAI) tool to carry out dietary nutritional assessments	Questionnaires assessing students’ knowledge of clinical assessment tools, functionality of new CAI, usefulness in improving nutritional assessment skills	Students completed simulations using the tool and found it easy to use, comprehensive and detailed in its content; however, they wished it had a search engine integrated and it lacked the ability to extrapolate the data to a statistical software Compared to other assessment tools students found it to be more intuitive and ‘more complete in the area of diagnosis’^18^ New tool was useful and 97.7% found it helped in identifying diseases and monitoring patients’ progress Overall score of 8.28 on scale of 1–10 for usefulness, functionality, and applicability	5/10
Bauer and Bai,^19^ 2015, Montclair State University, New Jersey, USA	34 students completing Master's in Nutrition and Food Science with a concentration on nutrition education across 2010 and 2011 cohorts	Interactive course on cultural competence exposing students to theoretical and practical knowledge about cultural competence	Pre‐ and post‐test comparison using IAPCC‐R 2002 and the five constructs from Campinha–Bacote model	Total competence score improved from ‘culturally aware’ (score of 68.7 at pre‐) to ‘culturally competent’ (score of 78.7 at post‐), *p* < 0.001 Scores for each construct of the model also improved after completion of the course, *p* < 0.001, except for desire *p* = 0.09 Students perceived that a course providing multiple interactive activities addressing constructs of this model was very useful Journals kept by students allowed the instructor to observe trends or changes in student opinions and reactions. For example: ‘I did not cringe or squirm (as I thought I might) when I read about the pig's throat being slit because I began to understand the practice as something very special and sacred to the Hmong’. I ask myself, ‘Who am I to judge the beliefs and practices of other people?’^19^	11/12
McCartan et al.,^20^ Monash University, Melbourne, Australia	22 first‐year Bachelor of Nutrition Science students. Mean age (SD) was 20 (7) years. 77% had previous experience meeting an Australian Aboriginal person and 73% had received Indigenous education at school	Evaluation surveys incorporating the CCMT to measure students’ self‐rating of cultural capability before and after exposure to Aboriginal health curriculum	Pre‐ and post‐comparison of survey using 25‐item CCMT measuring cultural capability	Students’ total CCMT scores increased significantly from T1 to T2 surveys (*p* = .001). Differences in CCMT scores between T1 and T2 were significant in five out of 25 items (*p* < .005). Two of these were in the respect learning domain and the other three were in communication, reflection, and advocacy learning domains. No items in the safety and quality learning domain were significantly different from T1 to T2. A statistically significant increase was found from T1 compared to T2 (*p* = .020) for rating of importance of an Aboriginal health curriculum in nutrition education, with the T2 median of 4.93 out of a possible 5 showing almost complete agreement	11/12

*Note*: CanMEDS is a Canadian framework that identifies and describes the abilities physicians require to effectively meet the health care needs of the people they serve.

Abbreviations: CASP, Critical Appraisal Skills Programme; CBA, competency‐based assessment; CCMT, Cultural Capability Measurement Tool; IAPCC‐R, Inventory for Assessing the Process of Cultural Competence among Healthcare Professionals – Revised; NOMA, NOrwegian MAsters (track module on nutrition, human rights and governance).

An interactive course exposing students to theoretical and practical knowledge about cultural competence was implemented in the study by Bauer and Bai.^19^ They used the Campinha–Bacote model to design, implement and evaluate the effectiveness of this course along with a pre‐ and post‐test comparison using the Inventory for Assessing the Process of Cultural Competence among Healthcare Professionals – Revised (IAPCC‐R).^19^ The Campinha–Bacote model is described as a process made up of five components that healthcare staff must learn to develop in order to deliver high‐quality care to patients in culturally diverse environments.^21^ These five components are cultural awareness, cultural knowledge, cultural skill, cultural desire and cultural encounter. Total competence scores significantly improved from ‘culturally aware’ (score of 68.7 at pre‐test) to ‘culturally competent’ (score of 78.7 at post‐test) (*p* < 0.001) after the interactive course. Cultural desire was the only one out of five constructs not to increase significantly after the course.

Similarly, McCartan et al.^20^ evaluated the effect an Aboriginal health curriculum had on the cultural capabilities of a cohort of first‐year students enrolled in a Bachelor level nutrition science program. A survey was created incorporating a 25‐item Cultural Capability Measurement Tool (CCMT). This tool measures five learning domains including respect, communication, safety and quality, reflection and advocacy.^22^ Students completed the survey in Semester 1 (T1) before receiving the Aboriginal health curriculum, and again during Semester 2 (T2) after completing the curriculum. Students’ total CCMT scores increased significantly from the T1 to T2 surveys (*p* = 0.001) indicating an increase in rating of their cultural capabilities after exposure to the curriculum. They were also asked to assess their attitude towards the importance of an Aboriginal health module. A statistically significant increase was found from T1 compared to T2 (*p* = 0.020) for the rating of importance, with the T2 median of 4.93 out of 5 showing almost complete agreement.^20^

In Brazil, Sabatini et al.^17^ used portfolio construction and focus groups to assess knowledge of food and culture. Three focus groups were conducted 1 year after the development of the portfolios to assess if they had a long‐term effect on the student's knowledge. Students felt that the portfolio had been demanding but instilled a curiosity and autonomy for learning in them to seek more information on food and culture. Greater appreciation of eating as a social and cultural act was observed following the completion of the task. The opinions expressed in the portfolios and during focus groups 1 year later suggest that the portfolio had a lasting effect in promoting autonomy and reflection.^17^

Simper et al.^15^ utilised pre‐ and post‐test comparison of motivational interview training with a cohort of final year nutrition undergraduates assessing their competence in behaviour change counselling. Video recordings of student–client counselling sessions were taken at baseline and following completion of the training to observe changes in counselling technique. Six behaviours were coded and significant improvements were seen for each across the cohort. These included a decrease in closed questions and non‐adherent behaviour that hinder behaviour change, and also an increase in open questions as well as complex and simple reflections. Students also improved their talking to listening time ratio, allowing clients to elaborate more before moving on to the next question. Proficiency at baseline was below where it should have been for all clinician‐behaviour counts. At follow‐up, these were above beginning proficiency showing an improvement for all students to being on the way to becoming competent. Improvement was significant for all counts (*p* < 0.001).^15^

Post‐test only outcomes were used to measure effectiveness in the remaining studies. Marais et al.^16^ evaluated the effectiveness of a module in increasing knowledge and awareness around the relationship between human rights, governance and nutrition. The module lasted for 18 weeks, after which self‐reflection interviews were carried out with students.^16^ The interviews found that students gained insight and increased their competence level as nutritionists in different roles, including communicator, collaborator, manager, health and nutrition advocate, and professional.

In 2015, Garcia de Diego et al.^18^ carried out an evaluation of a new computer assisted instruction tool for nutritional assessment. Thirty student volunteers completed the simulations and found the new tool user friendly, with 97.7% of the students finding it helpful for identifying diseases and monitoring patients progress.

## DISCUSSION

This systematic literature review aimed to understand how CBA is implemented and evaluated in nutrition education. The assessments focused on the development of key skills, including motivational interviewing^15^ and nutrition assessment,^18^ changes to knowledge and attitudes on food and culture,^17^, ^20^ and self‐perceived development of communication, collaboration, management, advocacy, scholarship and professional capabilities.^16^ Our results show that there is limited empirical literature describing CBA in nutrition education, with only six studies identified compared to a similar review recently completed in dietetics that identified 37 studies.

Given that there are currently 57 undergraduate degrees and 38 postgraduate degrees accredited with the AfN, it is surprising to see a lack of published literature on credibility, dependability or feasibility of CBA approaches in nutrition. This finding highlights a lack of focus in nutrition education research. It was, however, reassuring to see the focus of assessment on issues such as behaviour change and cultural understanding in some of the included studies. Ensuring that nutritionists are culturally appropriate in their practice is vitally important as populations diversify across the world. An effective nutrition workforce not only understands cultural traditions and reasons for choosing foods, diets, and lifestyles, but also is able to critically examine how their own cultural beliefs, values and implicit biases impact on their practice. Addressing racism and discrimination is a challenge in the preparation of other healthcare professionals.^23^ The lack of published literature with respect to assessment supporting competency in the promotion of health and wellbeing, and on the food chain system, was concerning. This was similar to a recent review conducted on CBA in dietetics where the focus was on assessment of individual patient assessment and management skills.^5^ The focus on pre‐post knowledge and self‐rated capability also limits the strength of the assessment outcome evidence.

None of the included studies reported a programmatic approach to assessment of competence where competence was viewed holistically but rather focused on individual competencies. Given that competencies are ‘an observable ability … integrating … knowledge, skills, values and attitudes.’^2^ and that competence is demonstrating the ability to draw together different skills and attributes to perform in complex settings, there is a need to work towards a more holistic view of CBA in nutrition. This holistic view of CBA in nutrition would move assessment towards authentic tasks that replicate the work required in current and future practice and ensure that nutritionists are prepared to be at the forefront of improving population health and nutrition.

The findings may appear to infer that nutrition education programs across the world are not implementing CBA. This was not the intention of the review but rather highlights the lack of published research in nutrition education. It is possible that nutrition degrees have robust CBA. However, because the scholarship of teaching and learning is in its infancy in nutrition, such work has not been published. We encourage educators to evaluate and publish their work, especially those implementing programmatic approaches that are positioned as best‐practice in CBA. This study is limited given the inclusion of English only studies, with studies from non‐English speaking countries not being represented.

Preparation of nutritionists for the work and jobs that will support improvements to population health and nutrition is a key part to reducing disease burden. Although competency standards have been articulated for this profession, this literature review provides evidence of a limited number of peer‐reviewed research papers in assessments of performance against these competency standards. There is a need to encourage the development of a program of research that supports the development and evaluation of credible, dependable and feasible CBA systems in nutrition education, or other dietetics or health profession assessments that may be applicable. This system will provide confidence that nutritionists are competent to meet current and future workforce needs.

## CONFLICT OF INTERESTS

The authors declare that there are no conflict of interests.

## AUTHOR CONTRIBUTIONS

All authors conceptualised the study. SOD was responsible for the study searches. LR supported the database search. CP was responsible for duplicate screening of abstracts. SOD and LR were responsible for data extraction. SOD, LR and CP were responsible for evidence synthesis. SOD drafted the manuscript. LR and CP contributed to manuscript preparation.

## TRANSPARENCY DECLARATION

The lead author affirms that this manuscript is an honest, accurate and transparent account of the study being reported. The reporting of this work is compliant with PRISMA guidelines. The lead author affirms that no important aspects of the study have been omitted and that any discrepancies from the study as planned (submitted for registration with PROSPERO: CRD42021237951) have been explained.

### PEER REVIEW

The peer review history for this article is available at https://publons.com/publon/10.1111/jhn.12946

## Supporting information

Supporting information.

Supporting information.
