# Supplementary material for: Competency‐based assessment in nutrition education: A systematic literature review
Source: J Hum Nutr Diet. 2021 Sep 19;35(1):102–11. doi: 10.1111/jhn.12946 (PMC9290644; doi:10.1111/jhn.12946)
Supplement: Supplementary file 1 — Supporting information. [file JHN-35-102-s002.pdf]

NutriPD
Growing Professional
Competence in Nutrition

Systematic Review Search Protocol

A systematic review of competency-based assessment (CBA) in nutrition and dietetics education.

Aim

The aim of this review is to understand what has been done in CBA in nutrition and dietetics education, and to evaluate students and educators understanding of the core competencies and their assessments.

Research question

1. Main (PICO)- How has CBA developed within nutrition/dietetics programs and what measures have been used to assess competency of nutritionists/dietitians?
2. Sub questions:
   a. How well do the assessment tools identified in the included studies align with the Association for Nutrition's competency standards in the UK?
   b. How well is the role of CBA understood?

Criteria for inclusion

Types of studies

Original research only to be considered. No editorial articles, reports, etc. to be included.

Timeline

No time limit will be placed on search initially.

Types of outcome measures

Outcome data will include:

- Using/development of CBA
- Opinion on CBA
- Pre- and post-competency analysis after implementation

Types of participants

Participants will be adults aged 18 years and above who are involved in nutrition or dietetics third level education or assessment (students, graduates, educators/assessors).

Types of interventions

Interventions of interest:

- Implementing CBA
- Evaluating CBA (feasibility point of view)

Exclude:

-Children

-Primary/secondary level education

These inclusion and exclusion criteria are summarised in Table 1 below.

Table 1: Inclusion and exclusion criteria for SLR on development or use of CBA in nutrition and dietetics education

PICO	Inclusion	Exclusion
Population	Humans<br>Adults ≥18 years<br>Students/graduates or educators/assessors of 3rd level nutrition/dietetics	Animals<br>Children<br>Courses on nutritional therapy<br>Degree programs less than level 8?
Intervention	Implementation of CBA<br>Evaluation of CBA	No mention of CBA or its evaluation
Comparator	N/A	-
Outcome	Opinion on CBA/Analysis of effectiveness/pre -& post-analysis of competency	No measure of competency analysis
Study design	Primary research<br>English language only	All other study designs and non-English publications

Search Term development

In order to select key words/search terms and identify appropriate databases for the SLR an initial search of electronic databases will be conducted (Phase I) using all potential key word terms (initial search terms). A second search using limited keywords/terms will be completed by the Lead Researcher using most appropriate databases and the number of hits will be recorded (Phase II). Key words, search criteria and databases will be discussed with the research team. A database search will be carried using suggested keywords and databases with hits recorded (Phase III). A search of finalised databases and keywords will be carried out and citations exported to Endnote library. A print screen of the search will be saved and recorded (Phase IV).

Phase 1: Initial search terms

Concept: Competence

Synonyms: Capability, competency, ability, capacity, proficiency, knowledge, skill, expertise.

Concept: Assessment

Synonyms: Evaluation, rating, appraisal, analysis, opinion, measure

Concept: Nutritionist

Synonyms: Dietitian, dietician, nutrition, dietetics

Concept: Implementation

Synonyms: Application, administration, performance, execution

Concept: Development

Synonyms: Advancement, evolution, improvement, progress, refinement

Databases identified

CINAHL

Pub Med

Science Direct

Web of Science

Abbreviations:

CBA- Competency-based assessment

CBME- Competency-based medical education

CC- Core competencies

EONDP- Education of Nutrition and Dietetics Practitioners

WBL- Work-based learning

Search Strategy: Phase II

Limited search terms and hits recorded

CINAHL Search Strategy		Pub Med Search Strategy	Hits
Competence* AND (nutrition OR dietetic)	937	Competence* AND (nutrition OR dietetic)	2,451
Competenc* AND (nutrition OR dietetic)	1,152	Competenc* AND (nutrition OR dietetic)	3,114
Competenc* AND (nutrition OR dietetic) AND assessment	210	Competenc* AND (nutrition OR dietetic) AND assessment	657
Competenc* AND (nutrition OR dietetic) AND development	230	Competenc* AND (nutrition OR dietetic) AND development	788
Competenc* AND (nutrition OR dietetic) AND develop*	322	Competenc* AND (nutrition OR dietetic) AND develop*	1,190
Competenc* AND (nutrition OR dietetic) AND implement*	85	Competenc* AND (nutrition OR dietetic) AND implement*	265

Science Direct Search Strategy	Hits	Web of Science Search Strategy	Hits
Competence AND (nutrition or dietetic)	11,978	Competence* AND nutrition OR dietetic	772
Competence AND (nutrition OR dietetic) AND assessment	6,229	Competenc* AND (nutrition OR dietetic)	1,091
Competence AND (nutrition OR dietetic) AND develop	9,682	Competenc* AND (nutrition OR dietetic) AND assessment*	194
Competence AND (nutrition OR dietetic) AND assess AND implement	3,030	Competenc* AND (nutrition OR dietetic) AND development	362
Competence AND (nutrition OR dietetic) AND assess NOT Animal	**4,837**	Competenc* AND (nutrition OR dietetic) AND develop*	508
Competency AND (nutrition OR dietetic) AND develop AND assess NOT animal	5,274	Competenc* AND (nutrition OR dietetic) AND implement*	114
Competency AND (nutrition OR dietetic) AND developing NOT animal NOT patient	**3,389**		

Notes

Science Direct: Use of wild cards are not supported in advanced search i.e. *

Search history section not available, therefore searching results of a number of previous searches with OR/AND not available e.g. S1 OR S2 OR S3

Web of Science:

Databases: institute available or manually select

Topic: searches article title, abstracts and keywords

Timespan: select years of search

Truncation *: wild card, can use *before/after* word or *both*

Proximity: “quotation marks” return exact phrase, NEAR/X. X being number of words term occurs near.

Need to be more specific for relevant info – consider using filters such as publication/article type, year, etc

Phase III

Further limited key words and database search following discussion with research team.

CINAHL Search Strategy		Pub Med Search Strategy	Hits
Competenc* AND (nutrition OR dietetic)	1,152	Competenc* AND (nutrition OR dietetic)	3,114

Science Direct Search Strategy	Hits	Web of Science Search Strategy	Hits
Competence AND (nutrition OR dietetic) [abstract- competence OR competency OR competencies]	1,669	Competenc* AND (nutrition OR dietetic)	1,091

Phase IV Database search

Search Terms

Competenc* AND (nutrition OR dietetic)

Database: PubMed

Saving a search in Pub Med

1. Sign in to My NCBI
2. Run a search and view search results
3. Click **Advanced** search button. The search history screen appears.
4. Click the number of the search you wish to save e.g. #1 under search icon. A dropdown menu appears, click save in My NCBI.
5. Enter **Name** of search and click save. Search will be saved in My NCBI.
6. To re-run search click My NCBI, saved searches, click search name, Pub Med runs the search and displays results screen. Can re-run search at any time.

To export search from Pub Med to EndNote

1. Open EndNote library
2. Following a search check the boxes of each article and check the **send to** box.
3. In the drop down **choose destination** column check the **citation manager** box
4. Create file

Database: Science Direct

Sign in via institution: search Galway Mayo Institute of Technology and click login

Click **Advanced** search

NOTE * wildcards not supported

Boolean operators use characters and connecters to assist in getting more accurate results. Some databases have their own rules. See below for Science Direct.

Database: Web of Science

Error on login

Database: CINAHL

Boolean/Phrase:

Publication date:

Search results:

Boolean/Phrase:

Search results

Saving a search in CINAHL

7. Run a search and view search results
8. Click **Search History** link, then click the **Save Searches/Alerts** link. The saved searches/Alerts screen appears. If you have not signed into MY EBSCOhost, you will be prompted to do so.

9. Enter a **Name** and **Description** for the search
10. In the **Save Search As** field, select the following: Saved Search (permanent)
11. To save the search, click **Save**

Retrieving saved search
Click ‘Folder’ icon (bar on top of page)
Left side My Folder option – click ‘Saved Searches’
Under My Folder: Saved Searches
Click Preliminary SLR_SOD
Click Retrieve saved search
Print search history
Copy and paste to doc

Exporting a search:
Click dropdown on ‘share’ icon
Scroll to ‘Export results’
Click ‘E-mail a link to download exported results’
Export manager, insert email details and click send
A link to retrieve exported items will be e-mailed to. Please retrieve them within 168 hours of receipt.

Coding:

1st Pass- All exported citations were coded based on their title using the Hierarchy of exclusion detailed below. (February 2020)

Hierarchy of Exclusion-

1. Duplicates - D
2. Not a population of interest - NP
3. Not a study design of interest (i.e. review paper) – NS
4. Not an intervention of interest - NI
5. Not an outcome of interest – NO
6. Not in English language - NE
7. Miscellaneous

2nd Pass- The included papers from 1st Pass were then coded based on abstracts.

3rd Pass- As over 150 papers were included after 2nd Pass, a decision was made to focus on ‘Nutrition’ education only. This brought the new total to 51 papers included. Full papers will now be read and coded based on the hierarchy for inclusion or exclusion.

*Updated search of databases using the same search procedure will be completed by two of the research team and abstracts will be checked in duplicate in March 2021. Following this a discussion will be had by the research team and any manuscripts meeting the inclusion criteria will be put forward for data analysis and extraction.

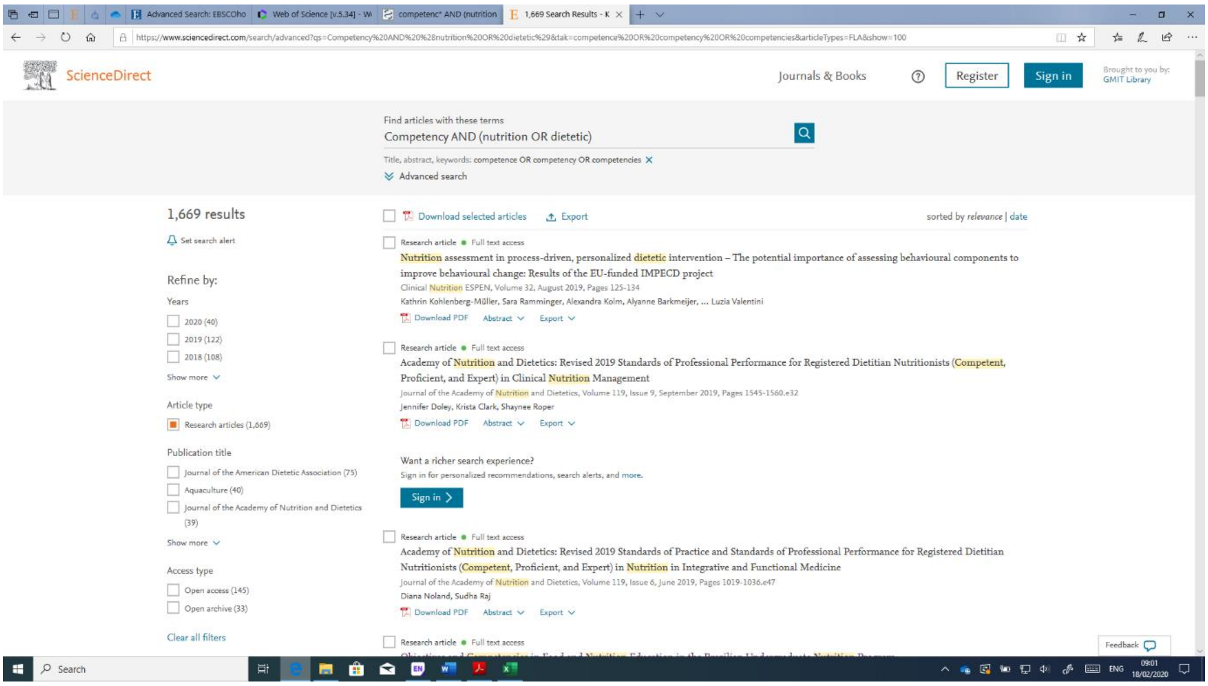

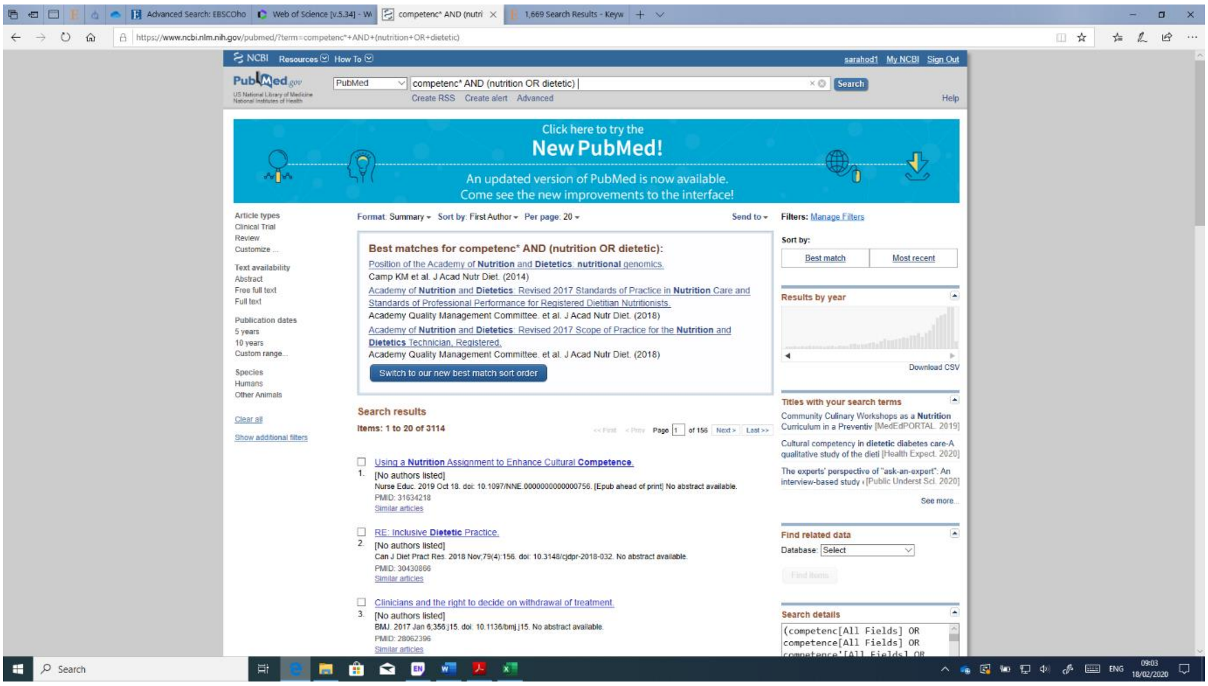
Advanced Search: EBSCOhost
Web of Science [v.5.34] - We
competenc* AND (nutri
1,669 Search Results - Keyw
https://www.ncbi.nlm.nih.gov/pubmed/?term=competenc*+AND+(nutrition+OR+dietetic)
NCBI Resources How To
sarahod1 My NCBI Sign Out
PubMed.gov
US National Library of Medicine National Institutes of Health
PubMed
competenc* AND (nutrition OR dietetic)
Search
Create RSS Create alert Advanced
Help
Click here to try the
New PubMed!
An updated version of PubMed is now available.
Come see the new improvements to the interface!
Article types
Clinical Trial
Review
Customize ...
Text availability
Abstract
Free full text
Full text
Publication dates
5 years
10 years
Custom range...
Species
Humans
Other Animals
Clear all
Show additional filters
Format: Summary Sort by: First Author Per page: 20
Send to
Best matches for competenc* AND (nutrition OR dietetic):
Position of the Academy of Nutrition and Dietetics: nutritional genomics.
Camp KM et al. J Acad Nutr Diet. (2014)
Academy of Nutrition and Dietetics: Revised 2017 Standards of Practice in Nutrition Care and Standards of Professional Performance for Registered Dietitian Nutritionists.
Academy Quality Management Committee. et al. J Acad Nutr Diet. (2018)
Academy of Nutrition and Dietetics: Revised 2017 Scope of Practice for the Nutrition and Dietetics Technician, Registered.
Academy Quality Management Committee. et al. J Acad Nutr Diet. (2018)
Switch to our new best match sort order
Search results
Items: 1 to 20 of 3114
<< First < Prev Page 1 of 156 Next > Last >>
1. Using a Nutrition Assignment to Enhance Cultural Competence.
[No authors listed]
Nurse Educ. 2019 Oct 18. doi: 10.1097/NNE.0000000000000756. [Epub ahead of print] No abstract available.
PMID: 31634218
Similar articles
2. RE: Inclusive Dietetic Practice.
[No authors listed]
Can J Diet Pract Res. 2018 Nov;79(4):156. doi: 10.3148/cjdpr-2018-032. No abstract available.
PMID: 30430866
Similar articles
3. Clinicians and the right to decide on withdrawal of treatment.
[No authors listed]
BMJ. 2017 Jan 6;356:j15. doi: 10.1136/bmj.j15. No abstract available.
PMID: 28062396
Similar articles
Filters: Manage Filters
Sort by:
Best match Most recent
Results by year
Download CSV
Titles with your search terms
Community Culinary Workshops as a Nutrition Curriculum in a Preventiv [MedEdPORTAL. 2019]
Cultural competency in dietetic diabetes care-A qualitative study of the dieti [Health Expect. 2020]
The experts' perspective of "ask-an-expert": An interview-based study [Public Underst Sci. 2020]
See more...
Find related data
Database: Select
Find items
Search details
(competenc[All Fields] OR
competence[All Fields] OR
Search
ENG
09:03
18/02/2020

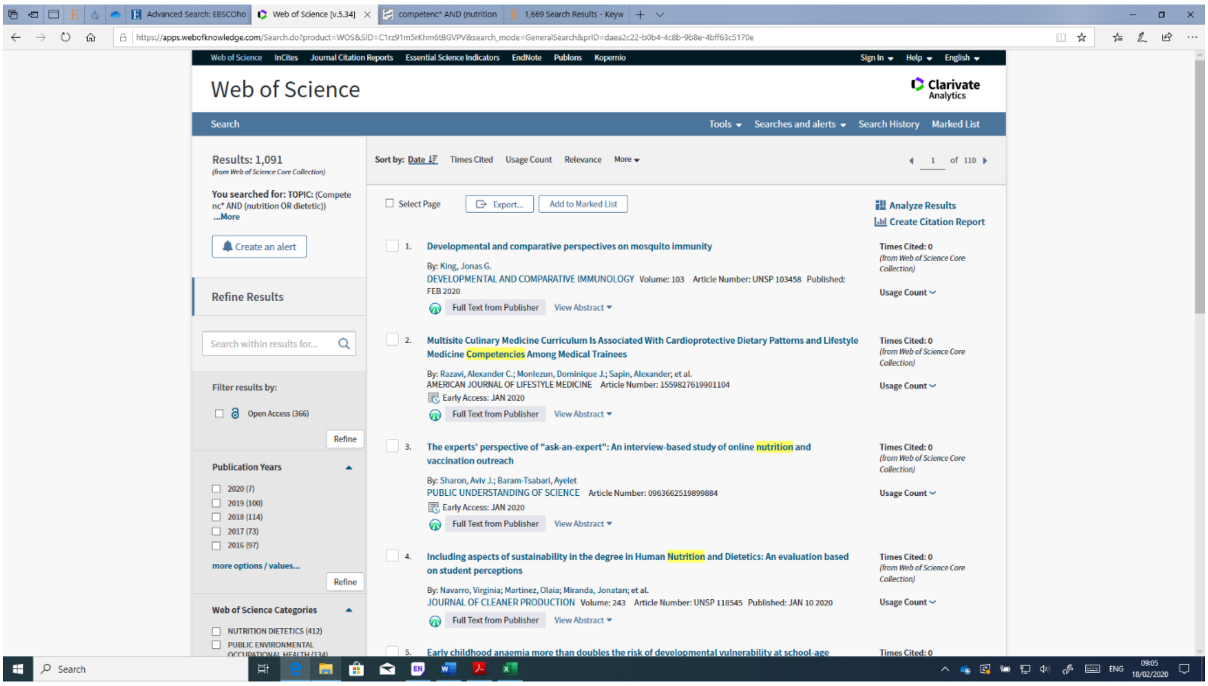
Advanced Search: EBSCOhost
Web of Science [v.5.34]
competenc* AND (nutrition
1,669 Search Results - Keyw
https://apps.webofknowledge.com/Search.do?product=WOS&SID=C1rz91m5rKhm6tBGVPV&search_mode=GeneralSearch&prID=daea2c22-b0b4-4c8b-9b8e-4bff63c5170e
Web of Science InCites Journal Citation Reports Essential Science Indicators EndNote Publons Kopernio
Sign In Help English
Web of Science
Clarivate Analytics
Search
Tools Searches and alerts Search History Marked List
Results: 1,091
(from Web of Science Core Collection)
You searched for: TOPIC: (Competenc* AND (nutrition OR dietetic))
...More
Create an alert
Refine Results
Search within results for...
Filter results by:
Open Access (366)
Refine
Publication Years
2020 (7)
2019 (100)
2018 (114)
2017 (73)
2016 (97)
more options / values...
Refine
Web of Science Categories
NUTRITION DIETETICS (412)
PUBLIC ENVIRONMENTAL OCCUPATIONAL HEALTH (134)
Sort by: Date Times Cited Usage Count Relevance More
1 of 110
Select Page
Export...
Add to Marked List
Analyze Results
Create Citation Report
1. Developmental and comparative perspectives on mosquito immunity
By: King, Jonas G.
DEVELOPMENTAL AND COMPARATIVE IMMUNOLOGY Volume: 103 Article Number: UNSP 103458 Published: FEB 2020
Full Text from Publisher View Abstract
Times Cited: 0
(from Web of Science Core Collection)
Usage Count
2. Multisite Culinary Medicine Curriculum Is Associated With Cardioprotective Dietary Patterns and Lifestyle Medicine Competencies Among Medical Trainees
By: Razavi, Alexander C.; Monlezun, Dominique J.; Sapin, Alexander; et al.
AMERICAN JOURNAL OF LIFESTYLE MEDICINE Article Number: 1559827619901104
Early Access: JAN 2020
Full Text from Publisher View Abstract
Times Cited: 0
(from Web of Science Core Collection)
Usage Count
3. The experts' perspective of "ask-an-expert": An interview-based study of online nutrition and vaccination outreach
By: Sharon, Aviv J.; Baram-Tsabari, Ayelet
PUBLIC UNDERSTANDING OF SCIENCE Article Number: 0963662519899884
Early Access: JAN 2020
Full Text from Publisher View Abstract
Times Cited: 0
(from Web of Science Core Collection)
Usage Count
4. Including aspects of sustainability in the degree in Human Nutrition and Dietetics: An evaluation based on student perceptions
By: Navarro, Virginia; Martinez, Olaia; Miranda, Jonatan; et al.
JOURNAL OF CLEANER PRODUCTION Volume: 243 Article Number: UNSP 118545 Published: JAN 10 2020
Full Text from Publisher View Abstract
Times Cited: 0
(from Web of Science Core Collection)
Usage Count
5. Early childhood anaemia more than doubles the risk of developmental vulnerability at school-age
Times Cited: 0
Search
ENG
09:05
18/02/2020

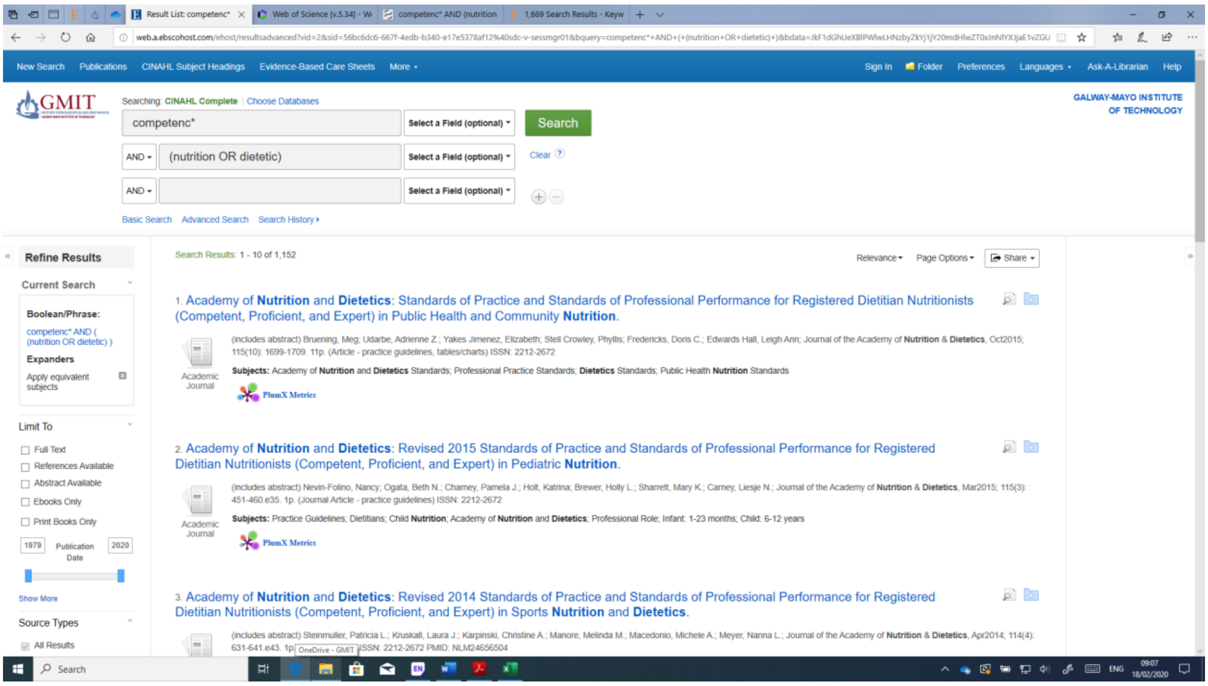
New Search
Publications
CINAHL Subject Headings
Evidence-Based Care Sheets
More
Sign In
Folder
Preferences
Languages
Ask-A-Librarian
Help
GMIT
Searching: CINAHL Complete
Choose Databases
competenc*
Select a Field (optional)
Search
AND
(nutrition OR dietetic)
Clear
Basic Search
Advanced Search
Search History
GALWAY-MAYO INSTITUTE OF TECHNOLOGY
Refine Results
Current Search
Boolean/Phrase:
competenc* AND ((nutrition OR dietetic))
Expanders
Apply equivalent subjects
Limit To
Full Text
References Available
Abstract Available
Ebooks Only
Print Books Only
1979
Publication Date
2020
Show More
Source Types
All Results
Search Results: 1 - 10 of 1,152
Relevance
Page Options
Share
1. Academy of Nutrition and Dietetics: Standards of Practice and Standards of Professional Performance for Registered Dietitian Nutritionists (Competent, Proficient, and Expert) in Public Health and Community Nutrition.
(includes abstract) Bruening, Meg; Udarbe, Adrienne Z.; Yakes Jimenez, Elizabeth; Stell Crowley, Phyllis; Fredericks, Doris C.; Edwards Hall, Leigh Ann; Journal of the Academy of Nutrition & Dietetics, Oct2015; 115(10): 1699-1709. 11p. (Article - practice guidelines, tables/charts) ISSN: 2212-2672
Subjects: Academy of Nutrition and Dietetics Standards; Professional Practice Standards; Dietetics Standards; Public Health Nutrition Standards
Academic Journal
PlumX Metrics
2. Academy of Nutrition and Dietetics: Revised 2015 Standards of Practice and Standards of Professional Performance for Registered Dietitian Nutritionists (Competent, Proficient, and Expert) in Pediatric Nutrition.
(includes abstract) Nevin-Folino, Nancy; Ogata, Beth N.; Charney, Pamela J.; Holt, Katrina; Brewer, Holly L.; Sharrett, Mary K.; Carney, Liesje N.; Journal of the Academy of Nutrition & Dietetics, Mar2015; 115(3): 451-460.e35. 1p. (Journal Article - practice guidelines) ISSN: 2212-2672
Subjects: Practice Guidelines; Dietitians; Child Nutrition; Academy of Nutrition and Dietetics; Professional Role; Infant: 1-23 months; Child: 6-12 years
Academic Journal
PlumX Metrics
3. Academy of Nutrition and Dietetics: Revised 2014 Standards of Practice and Standards of Professional Performance for Registered Dietitian Nutritionists (Competent, Proficient, and Expert) in Sports Nutrition and Dietetics.
(includes abstract) Steinmuller, Patricia L.; Kruskall, Laura J.; Karpinski, Christine A.; Manore, Melinda M.; Macedonio, Michele A.; Meyer, Nanna L.; Journal of the Academy of Nutrition & Dietetics, Apr2014; 114(4): 631-641.e43. 1p. ISSN: 2212-2672 PMID: NLM24656504
Search
ENG
09:07
18/02/2020

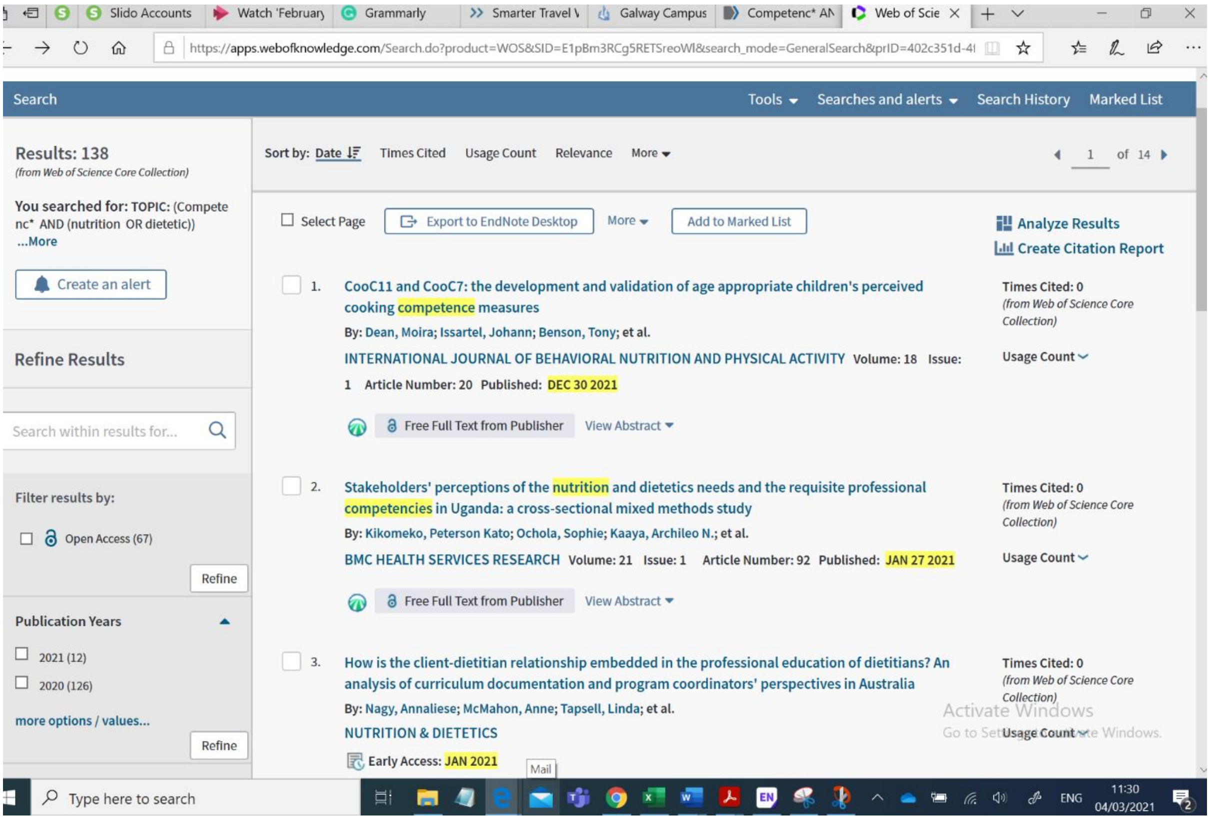
Slido Accounts
Watch 'February
Grammarly
Smarter Travel
Galway Campus
Competenc* AN
Web of Scie
https://apps.webofknowledge.com/Search.do?product=WOS&SID=E1pBm3RCg5RETSreoWl&search_mode=GeneralSearch&prID=402c351d-4
Search
Tools
Searches and alerts
Search History
Marked List
Results: 138
(from Web of Science Core Collection)
You searched for: TOPIC: (Competenc* AND (nutrition OR dietetic))
...More
Create an alert
Refine Results
Search within results for...
Filter results by:
Open Access (67)
Refine
Publication Years
2021 (12)
2020 (126)
more options / values...
Refine
Sort by: Date
Times Cited
Usage Count
Relevance
More
1 of 14
Select Page
Export to EndNote Desktop
More
Add to Marked List
Analyze Results
Create Citation Report
1. CooC11 and CooC7: the development and validation of age appropriate children's perceived cooking competence measures
By: Dean, Moira; Issartel, Johann; Benson, Tony; et al.
INTERNATIONAL JOURNAL OF BEHAVIORAL NUTRITION AND PHYSICAL ACTIVITY Volume: 18 Issue: 1 Article Number: 20 Published: DEC 30 2021
Free Full Text from Publisher
View Abstract
Times Cited: 0
(from Web of Science Core Collection)
Usage Count
2. Stakeholders' perceptions of the nutrition and dietetics needs and the requisite professional competencies in Uganda: a cross-sectional mixed methods study
By: Kikomeko, Peterson Kato; Ochola, Sophie; Kaaya, Archileo N.; et al.
BMC HEALTH SERVICES RESEARCH Volume: 21 Issue: 1 Article Number: 92 Published: JAN 27 2021
Free Full Text from Publisher
View Abstract
Times Cited: 0
(from Web of Science Core Collection)
Usage Count
3. How is the client-dietitian relationship embedded in the professional education of dietitians? An analysis of curriculum documentation and program coordinators' perspectives in Australia
By: Nagy, Annaliese; McMahon, Anne; Tapsell, Linda; et al.
NUTRITION & DIETETICS
Early Access: JAN 2021
Times Cited: 0
(from Web of Science Core Collection)
Usage Count
Activate Windows
Go to Settings to activate Windows.
Type here to search
ENG
11:30
04/03/2021

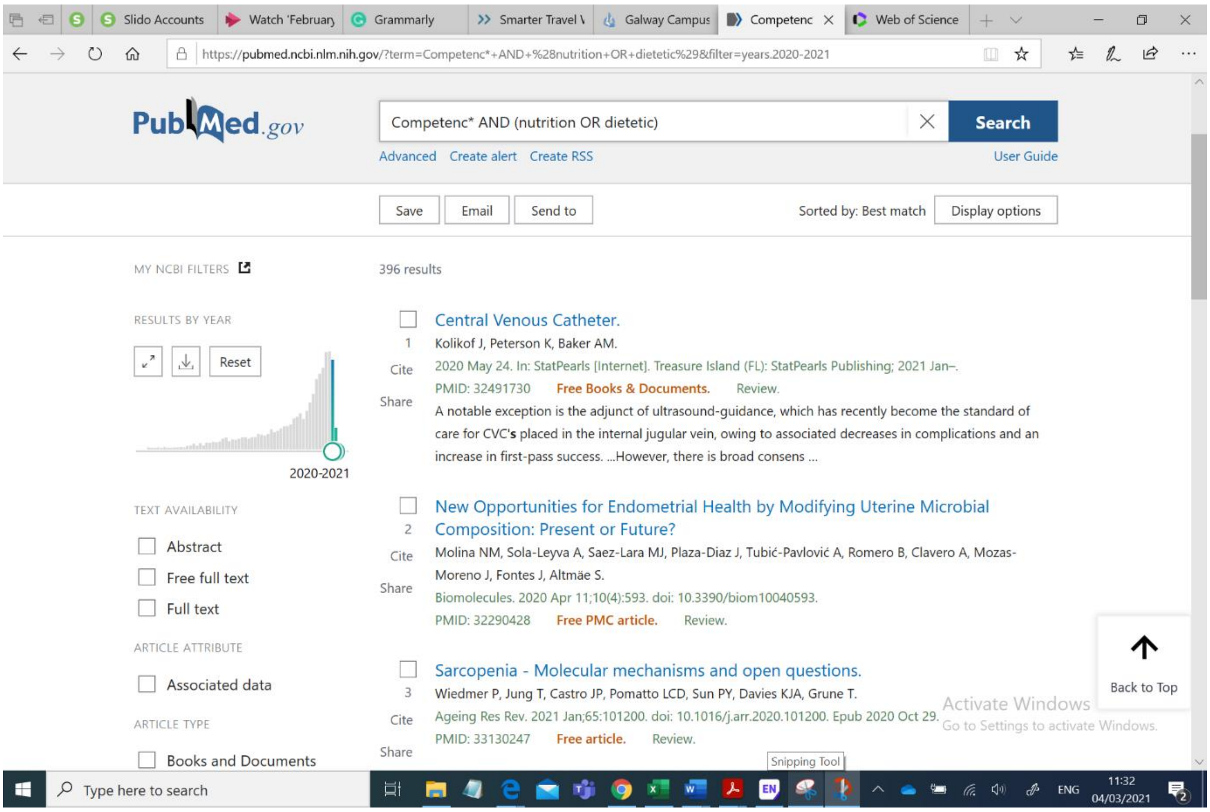
PubMed.gov
Competenc* AND (nutrition OR dietetic)
Search
Advanced Create alert Create RSS
User Guide
Save Email Send to
Sorted by: Best match Display options
MY NCBI FILTERS
396 results
RESULTS BY YEAR
Reset
2020-2021
Central Venous Catheter.
Kolikof J, Peterson K, Baker AM.
2020 May 24. In: StatPearls [Internet]. Treasure Island (FL): StatPearls Publishing; 2021 Jan–.
PMID: 32491730 Free Books & Documents. Review.
A notable exception is the adjunct of ultrasound-guidance, which has recently become the standard of care for CVC's placed in the internal jugular vein, owing to associated decreases in complications and an increase in first-pass success. ...However, there is broad consens ...
TEXT AVAILABILITY
Abstract
Free full text
Full text
New Opportunities for Endometrial Health by Modifying Uterine Microbial Composition: Present or Future?
Molina NM, Sola-Leyva A, Saez-Lara MJ, Plaza-Diaz J, Tubić-Pavlović A, Romero B, Clavero A, Mozas-Moreno J, Fontes J, Altmäe S.
Biomolecules. 2020 Apr 11;10(4):593. doi: 10.3390/biom10040593.
PMID: 32290428 Free PMC article. Review.
ARTICLE ATTRIBUTE
Associated data
Sarcopenia - Molecular mechanisms and open questions.
Wiedmer P, Jung T, Castro JP, Pomatto LCD, Sun PY, Davies KJA, Grune T.
Ageing Res Rev. 2021 Jan;65:101200. doi: 10.1016/j.arr.2020.101200. Epub 2020 Oct 29.
PMID: 33130247 Free article. Review.
ARTICLE TYPE
Books and Documents
Back to Top
11:32
04/03/2021

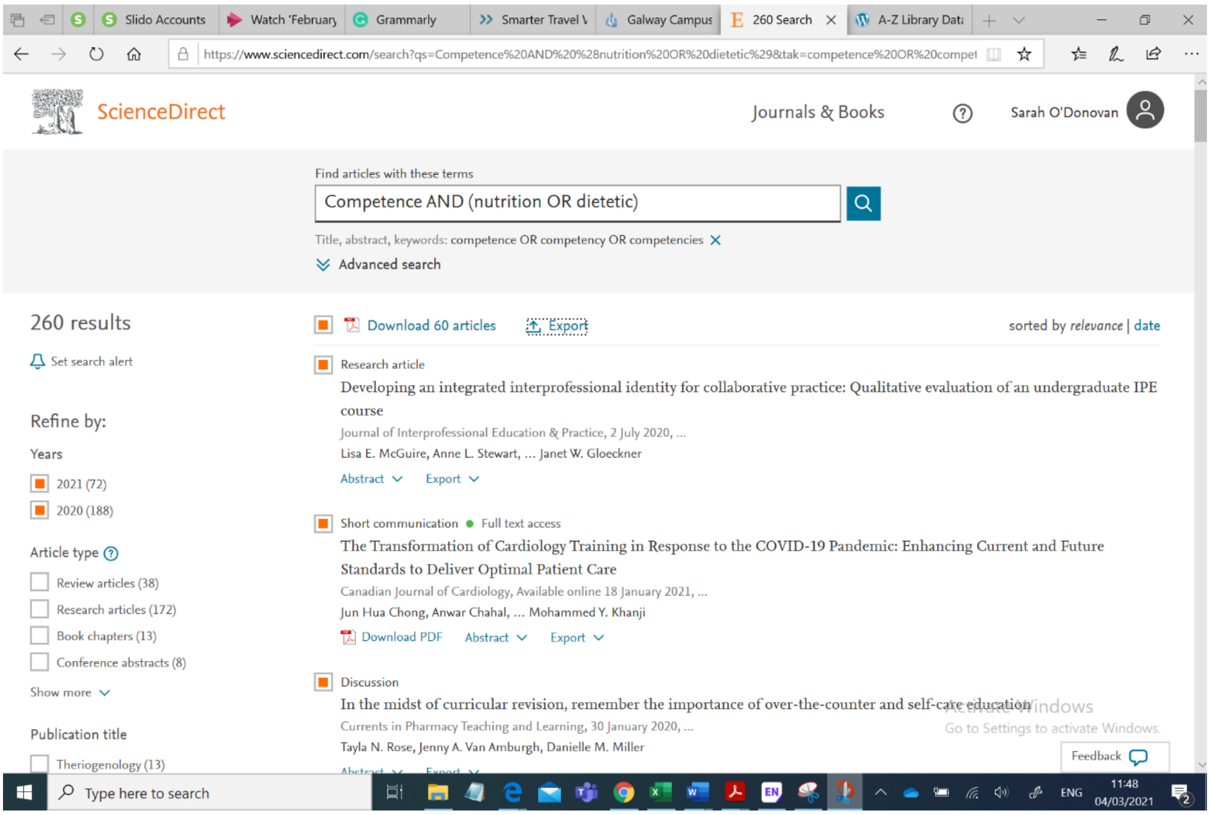
ScienceDirect
Journals & Books
Sarah O'Donovan
Find articles with these terms
Competence AND (nutrition OR dietetic)
Title, abstract, keywords: competence OR competency OR competencies
Advanced search
260 results
Set search alert
Download 60 articles Export
sorted by relevance | date
Refine by:
Years
2021 (72)
2020 (188)
Research article
Developing an integrated interprofessional identity for collaborative practice: Qualitative evaluation of an undergraduate IPE course
Journal of Interprofessional Education & Practice, 2 July 2020, ...
Lisa E. McGuire, Anne L. Stewart, ... Janet W. Gloeckner
Article type
Review articles (38)
Research articles (172)
Book chapters (13)
Conference abstracts (8)
Show more
Short communication Full text access
The Transformation of Cardiology Training in Response to the COVID-19 Pandemic: Enhancing Current and Future Standards to Deliver Optimal Patient Care
Canadian Journal of Cardiology, Available online 18 January 2021, ...
Jun Hua Chong, Anwar Chahal, ... Mohammed Y. Khanji
Download PDF Abstract Export
Discussion
In the midst of curricular revision, remember the importance of over-the-counter and self-care education
Currents in Pharmacy Teaching and Learning, 30 January 2020, ...
Tayla N. Rose, Jenny A. Van Amburgh, Danielle M. Miller
Publication title
Theriogenology (13)
11:48
04/03/2021
