# Supplementary material for: Competency‐based assessment in nutrition education: A systematic literature review
Source: J Hum Nutr Diet. 2021 Sep 19;35(1):102–11. doi: 10.1111/jhn.12946 (PMC9290644; doi:10.1111/jhn.12946)
Supplement: Supplementary file 2 — Supporting information. [file JHN-35-102-s001.pdf]

*Outline of the AfN's[a] Five Core Competencies and the Knowledge/Skills Expected to be Covered Under Each for an Associate Nutritionist (ANutr)[(11)]**

**Core Competency 1 - Science**	
**CC1a**	The human/animal body and its functions, especially digestion, absorption, excretion, respiration, fluid and electrolyte balance, cardiovascular, neuro-endocrine, musculoskeletal and haematological systems, immunity and thermoregulation, energy balance and physical activity
**CC1b**	Mechanisms for the integration of metabolism, at molecular, cellular and whole-body levels for either human or animal systems
**CC1c**	What nutrients are (including water & oxygen)
**CC1d**	Nature and extent of metabolic demand for nutrients
**CC1e**	How nutrients are used by the body (either human or animal) consequences of deficiency and assessment of nutritional status
**CC1f**	Non-nutrient components of foods, feeds and drinks that affect diet and health including alcohol for either human or animal systems.
**CC1g**	Nutrient analysis: calculating nutrient contents of foods, feeds and diets of an individual or group of individuals or animals, justifying choice of a method of dietary assessment for a specific stated purpose
**CC1h**	Digestion, absorption, transportation and storage of nutrients and non-nutrient components of foods or feeds for either human or animal systems
**CC1i**	Nutrition in health and disease, consequences of an unbalanced diet for either human or animal systems
**CC1j**	Nature of common conditions that require dietary manipulation or can affect physical activity, such as obesity, diabetes, hypertension, cardiovascular disease, cancer etc. for either human or animal systems
**CC1k**	How nutritional needs change with age, gender, physical activity, lifestyle etc. for either human or animal systems
**CC1l**	Ability to plan, conduct, analyse and report on investigations into an aspect of nutrition in a responsible, safe and ethical manner
**CC1m**	Ability to carry out sample selection and to ensure validity, accuracy, calibration, precision, replicability and highlight uncertainty during collection in accordance with the basic principles of good clinical practice

**CC1n**	Ability to obtain, record, collate, analyse, interpret and report nutrition-related data using appropriate qualitative and quantitative research and statistical methods in the field and/or laboratory and/or intervention studies, working individually or in a group, as is most appropriate for the discipline under study
**CC1o**	Prepare, process, interpret and present data, using appropriate qualitative and quantitative techniques, statistical programmes, spreadsheets and programs for presenting data visually
**CC1p**	Health research methods, dietary nutrition methodologies and nutritional epidemiology for either human or animal systems
**CC1q**	Theories of and development of practical skills in communication and learning
**Core Competency 2 - Food or Feed Chain**	
**CC2a**	Food or feed commodities (staple foods, main sources of key nutrients, novel sources etc.) within UK and/or internationally for either human or animal systems
**CC2b**	Effect on chemical composition and nutritional quality of food, feed and diet for either human or animal systems of:<br>- methods of food or feed production, preparation, preservation, fortification and format<br>- sources of food or feed supply<br>- methods of cooking and storage
**CC2c**	Familiarity with and/or development of practical skills involved in the methods to analyse the composition of foods or feeds
**CC2d**	Ability to formulate ideas and opinions concerning foods or feeds, nutrients, non-nutrient components of food and nutrition effectively and appropriately for either human or animal systems
**CC2e**	Understanding of issues associated with food or feed sustainability
**Core Competency 3 - Social/Behavioural**	
**CC3a**	Food or feed and nutrition and health policy (at global, national and local level) for either human or animal systems
**CC3b**	Significance of evaluation of nutrition in maintaining and driving public health agendas
**CC3c**	Factors that affect an individual’s, communities’ and population groups’ nutritional needs and practices for either human or animal systems

**CC3d**	Religious and cultural beliefs and practices that impact on food, nutrition and health
**CC3e**	Consideration of financial/social and environmental circumstances on diet and nutritional intake
**CC3f**	Theories and application of methods of improving health, behaviour and change for either human or animal systems
**CC3g**	Design and implementation of intervention projects and programmes, methods for monitoring and evaluating effectiveness and efficiency
**CC3h**	Theories of nutrition health education and nutrition health promotion (humans only)
**CC3i**	Ability to design/formulate a diet to meet a specification appropriate for a stated situation for an individual, human or animal, or group of humans or animals
**Core Competency 4 - Health/Wellbeing**	
**CC4a**	Principles and methods of measurement and estimation of energy balance; energy expenditure physical activity and fitness; body mass; body composition; how body mass and energy balance are controlled for either human or animal systems
**CC4b**	Theory and methods of investigating the dietary, nutrient and activity patterns of the general population, subgroups and the individual for either human or animal systems
**CC4c**	Scientific basis of the safety and health promoting properties of nutrients and non-nutrient components of food or feed, based on knowledge of the metabolic effects of nutrients, anti-nutrients, toxicants, additives, pharmacologically active agents (drugs); nutrient-nutrient interactions, nutrient-gene interactions, 'nutraceuticals', functional foods, and any other metabolically active constituents of foods or feeds and the diet
**CC4d**	Scientific basis for the measurement and estimation of nutritional requirements, dietary reference values for the general population for either human or animal systems
**CC4e**	Understanding the general principles underpinning, and strengths and limitations of, common methods of assessment of nutritional status including clinical, anthropometric, dietary, biochemical, physiological, and functional methods for either human or animal systems
**CC4f**	Understanding the general principles and methods associated with determining the

	efficacy, health attributes, health claims, safety, and legal aspects of foods, feeds, drinks and supplements for either human or animal systems
**CC4g**	Ability to recognise strengths and weaknesses in dietary, nutrition and health research methods, in order to understand the limitations of the scientific basis of nutritional knowledge for either human or animal systems
**CC4h**	Ability to integrate knowledge and understanding from a variety of sources to identify or propose solutions in one of the following areas: Improvement of human health or improvement of the welfare and/or productivity of animals or improvement of food production and sustainability
**Core Competency 5 – Professional Conduct**	
**CC5a**	Ethics and values of professions
**CC5b**	AfN Standards of Ethics, Conduct and Performance
**CC5c**	Legal context of nutrition practice; including current relevant legislation and guidelines to providing information to individuals
**CC5d**	Responsibilities and accountability in relation to the current national and international legislation, national guidelines, local policies and protocols and clinical/corporate Governance in relation to nutrition
**CC5e**	Can recognise the moral and ethical issues of investigation and appreciate the need for ethical standards and professional codes of conduct applicable to both interventional and observational studies
**CC5f**	The relevance of research governance frameworks
**CC5g**	Intellectual property issues

[a]AfN= Association for Nutrition

*reproduced with permission from the AfN
